# Mucinous Tubular and Spindle Cell Carcinoma of the Kidney: A Rare Renal Neoplasm—Case Report and Literature Review

**DOI:** 10.3390/reports8040189

**Published:** 2025-09-23

**Authors:** Ionuţ Burlacu, Mariana Aşchie, Mădălina Boşoteanu, Gabriela Izabela Bălţătescu, Alexandra Dinu

**Affiliations:** 1 Clinical Service of Pathology, “Sf. Apostol Andrei” Emergency County Hospital, 900591 Constanţa, Romania; burlacuionut82@yahoo.com (I.B.); aschiemariana@yahoo.com (M.A.); mbosoteanu@yahoo.com (M.B.); gabriela.baltatescu@univ-ovidius.ro (G.I.B.); 2 Institute of Doctoral Studies, Doctoral School of Medicine, “Ovidius” University of Constanţa, 900573 Constanţa, Romania; 3 Faculty of Medicine, “Ovidius” University of Constanţa, 900527 Constanţa, Romania; 4 Department of Anatomy, Academy of Medical Sciences of Romania, 030171 Bucharest, Romania; 5 Center for Research and Development of the Morphological and Genetic Studies of Malignant Pathology—CEDMOG, “Ovidius” University of Constanţa, 900591 Constanţa, Romania

**Keywords:** kidney neoplasms, rare renal tumors, mucinous tubular and spindle cell carcinoma, nephrectomy, immunohistochemistry, differential diagnosis

## Abstract

**Background and Clinical Significance:** Mucinous tubular and spindle cell carcinoma (MTSCC) is an uncommon subtype of renal cell carcinoma, representing 1–4% of epithelial renal tumors. It usually shows a low-grade morphology and indolent behavior, although sarcomatoid variants with an aggressive course have been described. Because of its overlap with papillary renal cell carcinoma (papRCC), sarcomatoid RCC, mesenchymal tumors, and oncocytic neoplasms, diagnosis requires the integration of imaging, histopathology, and immunohistochemistry. **Case Presentation:** We report a 71-year-old female who presented with a three-month history of right-sided lumbar pain and intermittent hematuria. Her laboratory tests were unremarkable. Contrast-enhanced CT revealed a well-circumscribed nodular lesion in the mid-portion of the right kidney, measuring 50 × 47 × 52 mm. The patient underwent right nephrectomy. Macroscopic findings revealed an encapsulated, yellowish-gray nodule (5.2 × 5 × 4 cm) without renal pelvis invasion. Microscopically, the tumor consisted of cuboidal- to spindle-shaped cells arranged in cords and tubular structures within a mucinous stroma, with focal necrosis and foamy macrophages. Immunohistochemistry showed positivity for CK19, CK7, EMA, PAX8, and AMACR, with a Ki-67 index <10%, while CD117, RCC, CD10, and chromogranin were negative. Together, the low Ki-67 proliferation index, absence of invasion, and low-grade histological architecture confirmed MTSCC of low malignant potential. At a five-year follow-up, the patient remained disease-free. **Conclusions:** MTSCC is a rare renal neoplasm that can be diagnosed by integrating clinico-radiological, histopathological, and immunophenotypic features. Molecular profiling may further distinguish MTSCC from papRCC and identify aggressive variants. Surgical excision remains the cornerstone of management, supported by vigilant long-term follow-up.

## 1. Introduction and Clinical Significance

Mucinous tubular and spindle cell carcinoma (MTSCC) represents an uncommon form of renal cell carcinoma (RCC), initially identified as a distinct pathological entity in the 2004 World Health Organization (WHO) classification of renal tumors and subsequently maintained in later editions, including the most recent 2022 update [1,2,3].

Histologically, MTSCC is characterized by elongated tubules and spindle-shaped cells embedded within a mucinous or myxoid stroma, typically with a low nuclear grade [4]. Morphological patterns, such as mucin-poor areas, focal papillary patterns, foamy macrophages, or necrosis, may complicate diagnosis and broaden the histologic spectrum [5,6]. Before its formal classification, tumors with overlapping features were frequently misdiagnosed as unclassified RCC, papillary RCC (papRCC) with atypical features, sarcomatoid carcinoma, or metanephric adenoma [7].

Radiologically, MTSCC often shows a distinctive profile. On computed tomography (CT) and magnetic resonance imaging (MRI), it typically exhibits slow, progressive contrast enhancement and intermediate-to-high T2 signal intensity with restricted diffusion. These features differ from the rapid wash-in and wash-out enhancement pattern of clear cell RCC and the homogeneous low enhancement typical of papRCC [8,9,10].

Immunohistochemically, MTSCC is usually positive for CK7, CK19, EMA, and AMACR (P504S), while CD10 expression is absent or weak. By contrast, papRCC typically shows strong and diffuse CD10 positivity [11]. PAX8 confirms renal origin, and a low Ki-67 index (<10%) supports its indolent nature [12]. Genomic studies have shown that MTSCC is characterized by multiple chromosomal deletions, while lacking the typical gains of chromosomes 7 and 17 that are commonly observed in papRCC [13,14,15,16].

Clinically, MTSCC is generally indolent and associated with favorable outcomes after surgical excision, although high-grade or sarcomatoid variants have been reported with a poor prognosis [2,5].

In this report, we describe a case of MTSCC located in the middle third of the right kidney, emphasizing clinical, imaging, histopathological, and immunohistochemical findings, together with molecular correlations, within the context of the contemporary literature.

## 2. Case Presentation

### 2.1. Clinical Presentation

A 71-year-old female patient with a known history of chronic obstructive pulmonary disease (COPD) and bronchial asthma was referred to our department with a three-month history of right-sided lumbar pain and intermittent hematuria. Due to persistent symptoms, the patient was admitted to the Urology Clinic of the “Saint Apostle Andrew” County Emergency Clinical Hospital of Constanţa for further diagnostic evaluation and specialized treatment.

**Figure 1:**
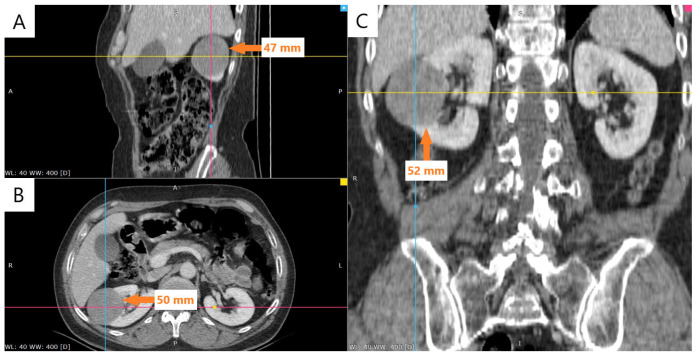
Contrast-enhanced CT in the venous phase (60 s), multiplanar reconstruction (MPR). (**A**) Axial image showing a well-defined nodular lesion in the mid-portion of the right kidney, measuring 47 mm (arrow). (**B**) Axial image with a maximum diameter of 50 mm (arrow). (**C**) Coronal image showing craniocaudal extension of 52 mm (arrow).

The laboratory profile was within normal limits. Abdominal and pelvic CT, performed in non-contrast and contrast-enhanced phases, showed a right kidney measuring 90 mm in bipolar diameter with a heterogeneous nephrogram. This aspect was attributed to the presence of a well-defined, nodular lesion located in the mid-portion of the kidney, protruding beyond the renal contour. The lesion appeared isodense on the non-contrast series and demonstrated low contrast enhancement, measuring approximately 50 × 47 mm in axial dimensions and 52 mm craniocaudally (Figure 1).

Based on clinical and imaging findings, a diagnosis of a right renal tumor was established. A surgical approach was deemed necessary, and the patient subsequently underwent a right open nephrectomy.

The patient’s preoperative and postoperative laboratory results are summarized in Table 1.

### 2.2. Pathological Findings

**Figure 2:**
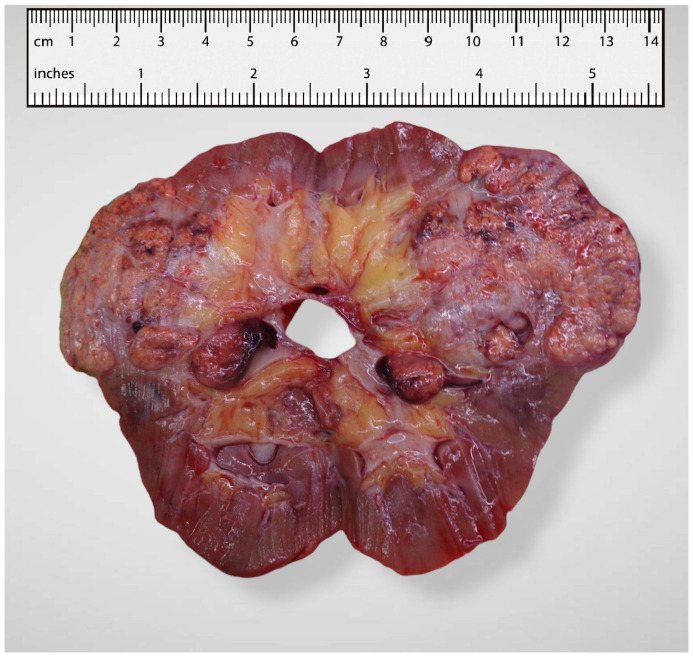
Gross pathological examination of the right nephrectomy specimen. The kidney measured 11 × 8 × 4 cm. In the mid-portion, extending toward the upper pole, a well-circumscribed encapsulated nodular lesion was identified, measuring 5.2 × 5 × 4 cm. The cut surface appeared to be yellowish-gray, with no evidence of invasion into the renal pelvis.

The nephrectomy specimen was received for gross pathological examination and measured 11 × 8 × 4 cm. At the mid-portion of the kidney, extending toward the upper pole, a well-circumscribed nodular lesion was identified, measuring 5.2 × 5 × 4 cm. The mass was encapsulated, displayed a yellowish-gray cut surface, and did not exhibit invasion into the renal pelvis (Figure 2).

**Figure 3:**
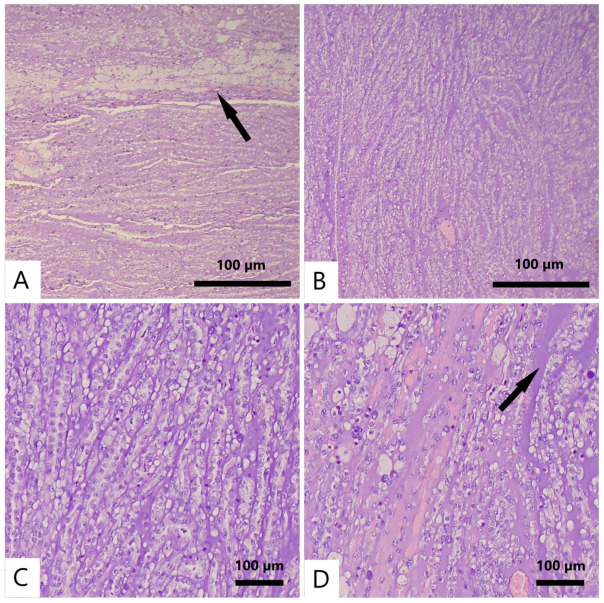
Representative microscopic features of the tumor (H&E). (**A**) Epithelial proliferation composed of cuboidal to oval cells with scant eosinophilic or clear cytoplasm and small nuclei showing mild pleomorphism, arranged in parallel cords and elongated, occasionally compressed tubular structures, with foamy macrophages at the periphery (arrow; ×5). (**B**) Anastomosing tubular structures (×5). (**C**) Tubules predominantly lined by flattened cuboidal cells (×10). (**D**) Tubular elements embedded within a mucinous stroma (arrow; ×10).

A microscopic examination of hematoxylin and eosin (H&E)-stained sections revealed an epithelial neoplasm composed of cuboidal to oval cells with scant, lightly eosinophilic or clear cytoplasm and small nuclei showing mild nuclear pleomorphism. Spindle-shaped cells lacking prominent nucleoli and atypical mitotic figures were also present. The tumor was arranged in parallel cords and elongated, occasionally compressed tubular structures embedded within a mucinous stroma. Focal necrosis and clusters of foamy macrophages were noted. There was no evidence of angiolymphatic, perineural, capsular, perirenal fat, renal sinus, renal vein, or ureteral invasion. Regional lymph nodes were not identified. Representative microscopic features are shown in Figure 3.

The definitive diagnosis was established following an immunohistochemical (IHC) analysis, the results of which are summarized in Table 2; the detailed immunohistochemistry panel is available in the Supplementary Materials (Table S1).

**Figure 4:**
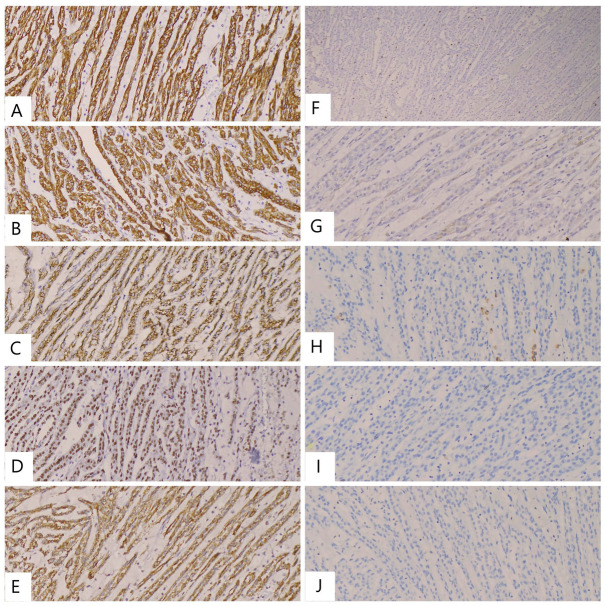
Immunohistochemical profile of the tumor. (**A**–**E**) Neoplastic cells showing positive staining for CK19 ((**A**), ×10), CK7 ((**B**), ×10), EMA ((**C**), ×10), PAX8 ((**D**), ×10), and AMACR ((**E**), ×10). (**F**) Ki-67 proliferation index <10% (≈5%) (×5). (**G**–**J**) Negative staining for RCC ((**G**), ×10), CD117 ((**H**), ×10), chromogranin ((**I**), ×10), and CD10 ((**J**), ×10).

The tumor cells demonstrated IHC positivity for CK19, CK7, EMA, PAX8, and AMACR. The Ki-67 proliferation index was quantified at <10% (approximately 5%). Conversely, IHC markers including CD117, chromogranin, RCC, and CD10 showed negative reactivity (Figure 4). These findings confirmed the final diagnosis of mucinous tubular and spindle cell carcinoma, pT1b, with low malignant potential, consistent with the initial microscopic impression.

The differential diagnosis included several renal neoplasms. Papillary RCC was ruled out due to the presence of a mucinous stroma and the absence of a distinctive papillary architecture. Sarcomatoid RCC was excluded based on the lack of marked nuclear pleomorphism, which is a hallmark of high-grade malignancy. Mesenchymal tumors were also considered; however, these are only rarely positive for cytokeratins. Oncocytoma and chromophobe RCC were excluded by their typical positivity for CD117, which was absent in this case. Neuroendocrine neoplasms were also part of the differential, but they were excluded by the lack of immunoreactivity for conventional neuroendocrine markers such as chromogranin [17,18].

At the most recent follow-up, five years after surgery, the patient remained asymptomatic and free of recurrence or metastasis.

## 3. Discussion

Mucinous tubular and spindle cell carcinoma accounts for only 1–4% of epithelial renal tumors, making it an uncommon subtype of renal cell carcinoma. The disease occurs predominantly in women, with a reported female-to-male ratio of 2–4:1 and is typically diagnosed at a median age of 50 years [5,16]. While most cases exhibit indolent behavior, aggressive forms—particularly those with sarcomatoid transformation—have also been reported [15,19]. Fewer than 100 cases of MTSCC are reported in the literature to date [20,21]. In a recent institutional series of 22 patients, the authors highlighted its female predominance, low proliferative index, and indolent clinical course [9], findings consistent with our case, which involved a female patient with low Ki-67 expression and an uneventful five-year follow-up.

In our patient, the lesion was well-circumscribed and located in the middle third of the kidney. CT imaging demonstrated minimal arterial enhancement with more pronounced venous phase uptake, a pattern consistent with previously described radiologic features of MTSCC [5].

A histopathological evaluation revealed the typical features of MTSCC, with cords and tubules of cuboidal cells blending into spindle cell areas within a mucin-rich stroma, along with focal necrosis and foamy macrophages [5,22]. Our case was staged as pT1b, in line with the early-stage tumors reported in both small institutional series and larger cohorts, in which pT1 disease consistently showed an indolent course [9,23]. This parallels the favorable outcome observed in our patient. The differential diagnosis included papillary RCC (excluded by the presence of mucinous stroma and absence of papillary architecture), sarcomatoid RCC (excluded by the lack of high-grade nuclear pleomorphism), mesenchymal tumors (rarely positive for cytokeratins), oncocytoma and chromophobe RCC (typically CD117-positive), and neuroendocrine neoplasms (excluded by negative chromogranin staining) [17,18].

Immunohistochemistry was decisive in establishing the diagnosis. Diffuse positivity for CK7, CK19, EMA, and AMACR supported MTSCC [18]. PAX8 confirmed renal origin, while CD117 and chromogranin were negative, excluding chromophobe/oncocytic and neuroendocrine tumors. The result of complete negativity for CD10 was helpful in ruling out papillary RCC [18]. The Ki-67 index was low (<10%), consistent with an indolent course [24].

In a series of nine MTSCC cases compared with ten papRCC cases, an immunohistochemical analysis showed 100% positivity for AMACR, CK7, and CK19 in MTSCC, but only 11% positivity for CD10 (versus 80% in papRCC), supporting CD10 as a useful discriminative marker [23]. Our case aligns with this pattern, showing MTSCC marker positivity and absent CD10 expression.

Genomic investigations further underline the distinction between MTSCC and papRCC. High-throughput sequencing and copy-number profiling have demonstrated recurrent chromosomal deletions together with mutations affecting genes such as NF2, CHEK2, and BRCA2 [16]. Characteristic molecular patterns include widespread losses (chromosomes 1, 4, 6, 8, 9, 13, 14, 15, and 22) in MTSCC, whereas papRCC more commonly displays gains of chromosomes 7 and 17 [13]. These chromosomal losses are observed even in high-grade or sarcomatoid cases, reinforcing the notion of MTSCC as a genetically distinct entity [5]. In advanced disease, alterations such as 1q gain and the homozygous deletion of CDKN2A/B have been linked to adverse outcomes [25,26]. The involvement of the MAPK and PI3K/AKT cascades has been proposed as a molecular basis for aggressive behavior [13,14,15,16], while Hippo pathway disruption—with bi-allelic inactivation of NF2, PTPN14, and SAV1 and increased nuclear expression of YAP1—has also been implicated as a potential oncogenic driver [27].

Taken together, MTSCC demonstrates a reproducible genomic profile, dominated by multiple chromosomal losses and lacking the gain patterns of papRCC. Additional alterations such as CDKN2A/B deletions and Hippo pathway dysregulation may underlie the rare aggressive phenotypes [5,13,16,25,26,27].

Clinically, most patients experience favorable outcomes after complete surgical excision [5,16]. However, sarcomatoid or high-grade variants are associated with a poor prognosis, often with a period of survival shorter than one year [15,17]. Because of their aggressive nature, sarcomatoid or high-grade MTSCC variants warrant more intensive surveillance compared with conventional cases [16,28]. In practical terms, this can involve CT or MRI performed every 3–6 months during the first two years, with annual imaging thereafter and follow-up extended beyond five years. Such a strategy is justified by the elevated risk of early relapse and metastatic spread [16,28]. Definitive management continues to rely on surgical resection, whereas systemic therapeutic approaches for advanced stages have not yet been standardized; recent reports suggest potential benefit from immune checkpoint blockade, particularly Ipilimumab plus Nivolumab, especially in cases with PD-L1 expression [15,29].

## 4. Conclusions

Mucinous tubular and spindle cell carcinoma is a rare and distinctive subtype of renal cell carcinoma. Accurate diagnosis requires the careful integration of clinical presentation, imaging, histopathological architecture, and immunohistochemical profile. Hallmark features—including a mucinous stroma, tubular and spindle cell arrangement, diffuse CK7/CK19/EMA/AMACR positivity, complete CD10 negativity, and a low Ki-67 proliferation index—are essential in distinguishing MTSCC from its histologic mimics.

Emerging evidence indicates that molecular profiling provides further insight to distinguish MTSCC from papillary RCC and recognize aggressive variants with less favorable outcomes. This biological heterogeneity emphasizes the importance of precise classification and long-term follow-up, even in low-grade cases.

We acknowledge that the present report is limited by its single-patient design and the absence of a comparative cohort, which inevitably restrict the generalizability of conclusions. Nevertheless, in this case, nephrectomy achieved complete disease control, with the patient remaining disease-free at five years, underscoring surgical excision as the cornerstone of therapy in localized disease and the necessity of prolonged surveillance.

## Figures and Tables

**Table 1:** Pre- and postoperative laboratory parameters of the patient.

Parameter	Measured Value at Hospital Admission	Postoperative Measured Value (Day 1)	Reference Range	Unit
**Blood biochemistry**				
Alanine aminotransferase	18	13	<33	U/L
Aspartate aminotransferase	20	18	<32	U/L
Creatinine	0.72	0.78	<1	mg/dL
Glucose	98	61	60–99	mg/dL
Urea	39	30		
**Complete Blood Count**				
Red blood cells	3.65	4.77	3.8–5.3	mil/µL
Hemoglobin	11.5	15.4	11.7–16	g/dL
Hematocrit	35.7	45	35–47	%
Mean corpuscular volume	97.8	94.3	81–101	fL
Mean corpuscular hemoglobin	31.5	32.3	27–34	pg/cell
Mean corpuscular hemoglobin concentration	32.2	34.2	31–36	g/dL
**White blood cell count**	5.7	6.32	4–10	K/µL
Lymphocytes	28.2	47.6	20–55	%
Monocytes	5.8	9.3	≤15	%
Neutrophils	63.9	37.1	45–80	%
Eosinophils	1.9	5.4	≤7	%
Basophils	0.2	0.6	≤2	%
Platelet count	228	176	150–450	K/µL
**Coagulation screening tests**				
International normalized ratio	1.03	-	0.8–1.2	
Partial thromboplastin time	32.2	-	<40	seconds
Quick time	12.7	-	-	seconds
**Urine biochemistry**				
Density	1015	-	1015–1025	
pH	6	-	4.8–7.4	
Leucocytes	negative	-	negative	
Nitrites	negative	-	negative	
Proteins	<10	-	<10	mg/dL
Glucose	undetectable	-	undetectable	
Ketone bodies	negative	-	negative	
Urobilinogen	normal	-	normal	
Bilirubine	negative	-	negative	
Eritrocytes	negative	-	negative	

**Table 2:** Panel of antibodies utilized in the immunohistochemical assessment.

Antibody	Result	Localization
CK7	Positive	Cytoplasmic
CK19	Positive	Cytoplasmic
EMA	Positive	Cytoplasmic
PAX8	Positive	Nuclear
AMACR	Positive	Cytoplasmic
CD117	Negative	–
RCC	Negative	–
CD10	Negative	–
Chromogranin	Negative	–
Ki-67	<10%	Nuclear

## Data Availability

The original data presented in the study are included in the article; further inquiries can be directed to the corresponding author.

## Supplementary Materials

Table S1: Detailed immunohistochemistry panel.

## Abbreviations

The following abbreviations are used in this manuscript:

CT	Computed tomography
H&E	Hematoxylin and eosin
IHC	Immunohistochemistry
MRI	Magnetic resonance imaging
MTSCC	Mucinous tubular and spindle cell carcinoma
papRCC	Papillary renal cell carcinoma
RCC	Renal cell carcinoma
WHO	World Health Organization
