# Supplementary material for: Mucinous Tubular and Spindle Cell Carcinoma of the Kidney: A Rare Renal Neoplasm—Case Report and Literature Review
Source: Reports (MDPI). 2025 Sep 23;8(4):189. doi: 10.3390/reports8040189 (PMC12643401; doi:10.3390/reports8040189)
Supplement: Supplementary file 1 [file reports-08-00189-s001.zip › reports-3862996-supplementary.pdf]

Table S1. Detailed immunohistochemistry panel

Antibody (Clone)	Isotype (Clonal Status)	Dilution	External Control (Protocol)	Protocol Source	Immunoreaction in Our Case (+/-)
CK19 (A53-B /A2.26/CellMarque)	Mouse Monoclonal Antibody	Ready to use	Bladder (Cytoplasmic); Colon carcinoma (Cytoplasmic); Colon (Cytoplasmic); Thyroid carcinoma (Cytoplasmic)	Ventana Roche	+ (cytoplasmic staining)
CK7 (SP52/Ventana)	Rabbit Monoclonal Primary Antibody	Ready to use	Lung, salivary gland, and breast tissues.	Ventana Roche	+ (cytoplasmic staining)
EMA (E29/Ventana)	Mouse Primary Monoclonal Antibody	Ready to use	Tonsil	Ventana Roche	+ (cytoplasmic staining)
PAX8 (MRQ-50/Cell/Marque)	Mouse Monoclonal Antibody	Ready to use	Ovarian carcinoma (non-mucinous carcinoma) (Nuclear); Thyroid carcinoma (Nuclear); Renal cell carcinoma (Nuclear)	Ventana Roche	+ (nuclear staining)
AMACR (13H4/Dako)	Monoclonal Rabbit	Ready to use	Prostate adenocarcinoma (Cytoplasmic)	Dako	+ (cytoplasmic staining)
Ki67 (MIB-1)	Mouse (monoclonal)	1:100	Tonsil	Dako	+ (nuclear staining—5%)
Chromogranin A (LK2H10)	Mouse (monoclonal)	Ready to use	Normal appendix	Ventana Roche	-
CD117 (Rb a Hu/Dako)	Polyclonal Rabbit Anti-Human	1:100	GIST	Dako	-
CD10 (SP67/Ventana)	Rabbit Monoclonal Primary Antibody	Ready to use	Tonsil	Ventana Roche	-
RCC (PN15)	Mouse Monoclonal Antibody	Ready to use	Kidney, Breast Thyroid, Renal Cell carcinoma	Ventana Roche	-
